# Prototype isochoric preservation device for large organs

**DOI:** 10.3389/fbioe.2024.1335638

**Published:** 2024-03-07

**Authors:** Alexandru Șerban, Gabriel Năstase, George-Andrei Beșchea, Ștefan-Ioan Câmpean, Cătălin Fetecău, Irinel Popescu, Florin Botea, Ion Neacșu

**Affiliations:** ^1^ Politechnica University of Bucharest, Bucharest, Romania; ^2^ Department of Building Services, Transilvania University of Brasov, Brasov, Romania; ^3^ Faculty of Mechanical Engineering, Dunarea de Jos University of Galati, Galati, Romania; ^4^ Center of General Surgery and Liver Transplantation, Fundeni Clinical Institute, Bucharest, Romania; ^5^ Criomec SA, Galati, Romania

**Keywords:** cryopreservation, isochoric, prototype development, organ preservation, subfreezing temperatures

## Abstract

This paper presents the design and prototype of a constant volume (isochoric) vessel that can be used for the preservation of large organs in a supercooled state. This prototype is a preliminary version of a more advanced design. The device consists of a cooling bath operated by a mechanical vapor compression refrigeration unit and an isochoric chamber made of stainless steel. The preservation of organs using supercooling technology in an isochoric chamber requires a continuous temperature and pressure monitoring. While the device was initially designed for pig liver experiments, its innovative design and preservation capabilities suggest potential applications for preserving other organs as well. The isochoric reactor may be used to accommodate a variety of organ types, opening the door for further research into its multi-organ preservation capabilities. All the design details are presented in this study with the purpose of encouraging researchers in the field to build their own devices, and by this to improve the design. We chose to design the device for isochoric supercooling as the method of preservation to avoid the ice formation.

## 1 Introduction

Life can be defined as a set of complex electrochemical reactions, also known as metabolism. Chemical reactions are temperature dependent. By reducing the metabolism by lowering the temperature, life can be extended. Cooling is known and used as a method of preservation, as it reduces the metabolism. In medicine, multiple thermal preservation techniques are used to preserve cells, tissues, and organs. Typically, cells and tissues are preserved through cryogenic methods, at temperatures below 0°C, the freezing point of water. A short-term alternative to cryopreservation is hypothermic preservation at temperatures between four°C and 10°C. Preservation of cells, tissues, and organs at a body temperature of 37°C is known as normothermic preservation.

The demand for transplantable organs is growing every day. Cooling an organ for preservation, for longer periods of time will offer surgeons the ability to do more transplants. The ability to preserve and transport the transplant organs for longer distances in a stable and safe environment will be an important achievement for medicine in general and for patients in particular. The biological matter contains large quantities of water, and since cryopreservation requires negative temperatures, ice formation acts as a damage mechanism. Lately, cryobiologists and engineers have focused on cryopreservation methods that avoid ice formation. One of the methods is isochoric freezing, which our research group examined using many types of biological matter. Isochoric freezing uses a metallic reactor with a constant volume in which the liquid freezes partially, leaving the biological matter with the possibility of being preserved in the liquid zone. A disadvantage of the method is the pressure generated inside the isochoric reactor, which is directly proportional to the preservation temperature and can vary from a few hundred bars to over 2000 bars. Another method of cryopreservation, more recent, is by using a supercooled fluid. Supercooling, expressed in degrees, usually means the number of degrees below the equilibrium melting temperature at which freezing of a liquid occurs. This method was and continues to be of great interest for our research group, but combined with isochoric preservation. A few results indicate that using an isochoric reactor can help improve the metastable supercooled state preservation method.

The design and use of an isochoric refrigerator for the preservation of biological matter at subfreezing temperatures in a supercooled state is introduced. The isochoric supercooling refrigerator concept draws from two earlier findings by our group. One is that monitoring the pressure in an isochoric chamber filled with aqueous fluid can detect the occurrence of freezing in the isochoric chamber. This finding is used for real-time control of isochoric supercooling in a feedback control loop. Pressure measurements can detect ice nucleation instantaneously because the information propagates with the speed of sound. The process of freezing is slow relative to the propagation of sound and heat. A control system was designed to stop the cooling and start the warming of the isochoric chamber the instant an increase in pressure is measured. Supercooling preservation continues once pressure measurements indicate that there is no more ice in the system. This facilitates supercooling preservation that is unlimited in time. The second finding is that an isochoric freezing system can contain multiple phases with different compositions separated by a boundary that transmits heat and pressure but not mass.

Preservation in a supercooled thermodynamic state is a way to reduce the metabolism of biological matter to subfreezing temperatures without the detrimental formation of ice. There is growing interest in preserving biological matter in a supercooled state for various applications in life sciences and food sciences. The idea of preserving biological matter in a supercooled state is not new (Luyet, 1952). A variety of techniques for supercooling preservation without ice were developed throughout the years. To list a few: emulsification (Luyet, 1952), elevated pressure (Bruinsma et al., 2015), electromagnetic fields (Monzen et al., 2005), cryoprotectant solutions at temperatures at which they do not freeze (Ishine et al., 1999; Puts et al., 2015), antifreeze proteins (Amir et al., 2004), partial freezing to mimic the survival of freeze-tolerant species (Ishine et al., 2000; Tessier et al., 2022), and deep-supercooling of large volumes with surface sealing with immiscible fluids (Huang et al., 2018). A series of valuable recent publications report the preservation of the liver in a supercooled state at subfreezing temperatures for extended periods of time (de Vries et al., 2019; Berendsen et al., 2014; de Vries et al., 2019; Huang et al., 2018; Bruinsma et al., 2015). Most of these technologies require the use of chemical additives that aid in maintaining the biological matter in a supercooled state. Also, the supercooling system methods listed above lack real-time control over the process of supercooling, i.e., the supercooling preservation process depends on the random statistical probability for ice nucleation.

In this paper, we introduce a new isochoric supercooling refrigerator that can be used in the modality of conventional isobaric refrigerators for the storage of large volumes and large numbers of biological items. The design is based on our earlier studies. Studies on the equilibrium thermodynamics of aqueous solutions in an isochoric system at subfreezing temperatures can be found in two fundamental publications (Rubinsky et al., 2005; Powell-Palm et al., 2020a). Earlier studies show that in an aqueous isochoric system, freezing yields an increase in pressure (Rubinsky et al., 2005; Perez, 2007; Powell-Palm et al., 2020a; Preciado and Rubinsky, 2010; Preciado, 2007). Therefore, measuring the pressure in an isochoric chamber is a simple way to detect the occurrence of freezing and the amount of ice in such a system (Rubinsky et al., 2005; Ukpai et al., 2017; Zhang et al., 2018; Rubinsky et al., 2020; Beschea et al., 2021). Pressure measurements are very sensitive to freezing in an isochoric chamber. For example, freezing 1% of the volume of water in the isochoric chamber results in an order of magnitude increase in pressure, from atmospheric 0.1 MPa–1 MPa (Rubinsky et al., 2005). The use of pressure measurements for real-time control of isochoric supercooling was described in our publications and patents, (Powell-Palm and Rubinsky, 2022; Consiglio et al., 2020). Specifically, “the rise in pressure accompanying ice formation triggers local heating of the container, which continues until the ice crystals have dissipated (as signaled by the dissipation of pressure) and supercooling can be resumed” (Powell-Palm and Rubinsky, 2022; Consiglio et al., 2020). Because in isochoric devices, pressure measurements can detect instant ice formations anywhere in the volume, if the increase in temperature during the melting of the ice crystals is brief, the biological matter can be preserved in a mostly supercooled state for an unlimited period of time, independent of the probability for ice nucleation (Powell-Palm and Rubinsky, 2022; Consiglio et al., 2020). The control system for isochoric supercooling that we have developed and the way it functions are possible only in an isochoric system. This control modality is demonstrated in this paper and makes isochoric supercooling preservation with pressure feedback, different from any of the previous supercooling preservation systems.

Research on supercooling in isochoric systems has shown that isochoric conditions increase the stability of metastable supercooled systems (Szobota and Rubinsky, 2006; Powell-Palm et al., 2020b; Consiglio et al., 2020; Consiglio et al., 2022; Câmpean et al., 2021). Isochoric supercooling was successfully used to preserve human cardiac microtissues (Powell-Palm et al., 2021) and in the food industry for the preservation of pomegranate juice (Bilbao-sainz et al., 2022). The work in this paper draws from our research on multiphase isochoric systems. The thermodynamics of multiphase isochoric systems were discussed in a number of publications (Perez, 2007; Powell-Palm et al., 2019; Rubinsky, 2022). The particular aspect of multiphase isochoric systems is that they are made of different phases separated by a boundary that can transfer pressure and heat, but not mass (Powell-Palm et al., 2019; Rubinsky, 2022; Powell-Palm and Rubinsky, 2022). This concept was used by us for a number of applications in food preservation by isochoric freezing (Bilbao-Sainz et al., 2019; Bilbao-Sainz et al., 2020; Bilbao-Sainz et al., 2020; Bilbao-Sainz et al., 2021)and by isochoric supercooling (Bilbao-sainz et al., 2022) and is used here in the design of a generic isochoric supercooling refrigerator.

## 2 Materials and methods

The isochoric vessel is a simple, cylindrical-shaped, constant-volume chamber designed to accommodate different pig organs. We designed the isochoric preservation device with the aim of using it in experiments with pig liver. The volumetry of a pig liver can vary with the pig’s age and weight but we considered as typical, a value of 2000 mL.

The chamber is instrumented with three RTD temperature sensors and a pressure transducer, all connected to a data monitoring and acquisition panel.

### 2.1 The isochoric vessel

**FIGURE 1:**
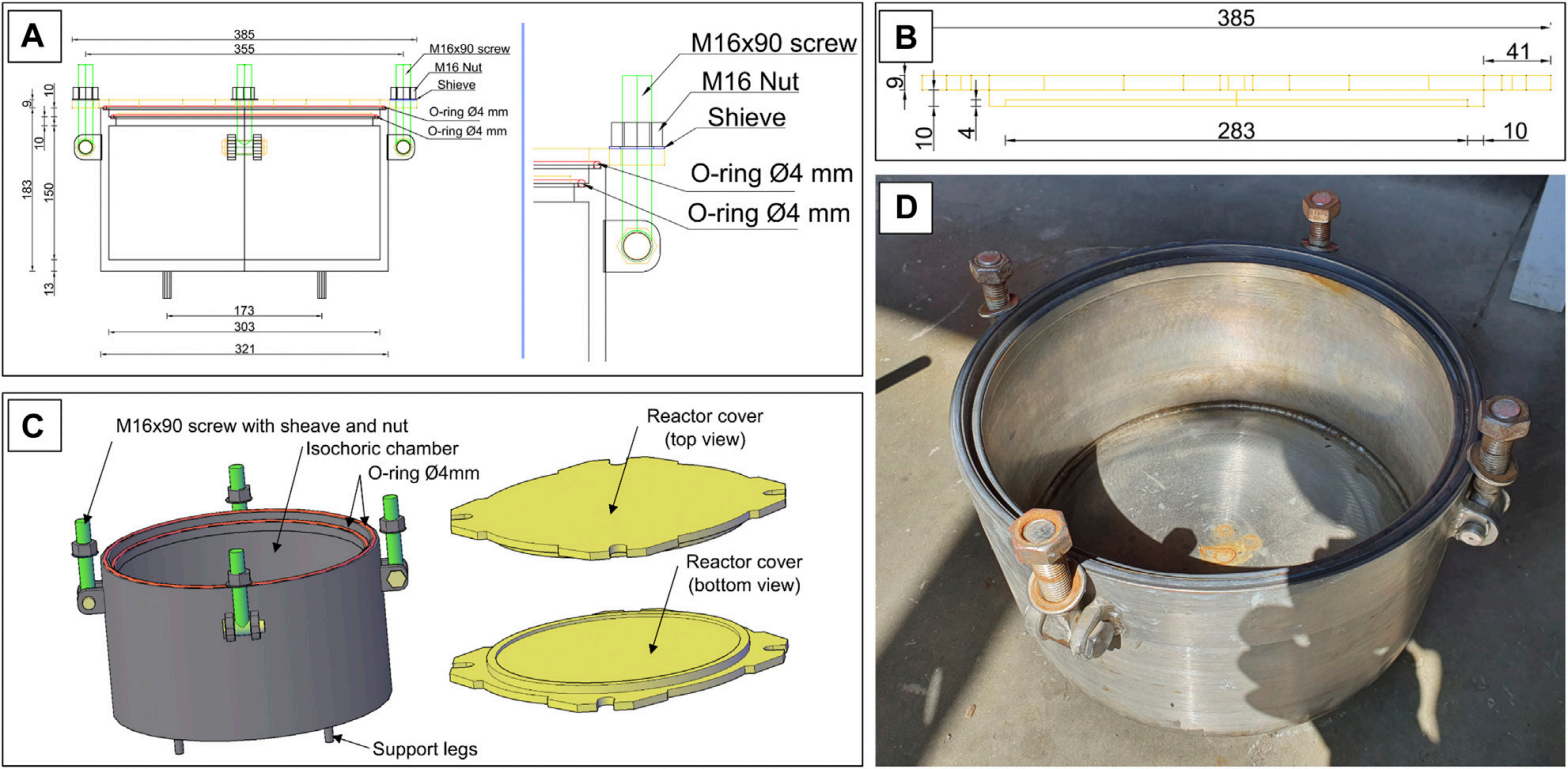
Wireframe section view of the reactor and a detail of the cap fastening system **(A)**; Reactor cover - side view **(B)**; A 3D model of the isochoric reactor and top and bottom views of the reactor’s cover **(C)**; A photograph with the inside view of the real reactor **(D)**.

The isochoric vessel is made entirely of stainless steel (austenitic stainless steel AISI 321 W1.4541 from ITALINOX ROMANIA SRL) and has an internal volume of 10.82 L. The reactor was made of a DN300 pipe (Ø303 × 9mm), to which a 13 mm stainless steel bottom (austenitic stainless steel AISI 321 W1.4541 from ITALINOX ROMANIA SRL) was welded. The reactor is placed on four 30 mm height, welded as shown in Figure 1. The purpose of these legs is to provide the necessary space to completely surround the isochoric reactor with the coolant (a mixture of water and ethylene glycol). At the top of the reactor, inside the pipe wall is a 4 × 2 mm channel that houses a rubber O-ring (Silicone rubber NBR70 O-ring, Dichtomatik - Freudenberg FST GmbH, Germany). Inside the reactor, at a distance of 10 mm from the top, is welded a 10 mm stainless steel ring (height) of 8 mm, which houses the second rubber O-ring (Silicone rubber NBR70 O-ring, Dichtomatik - Freudenberg FST GmbH, Germany).

**FIGURE 2:**
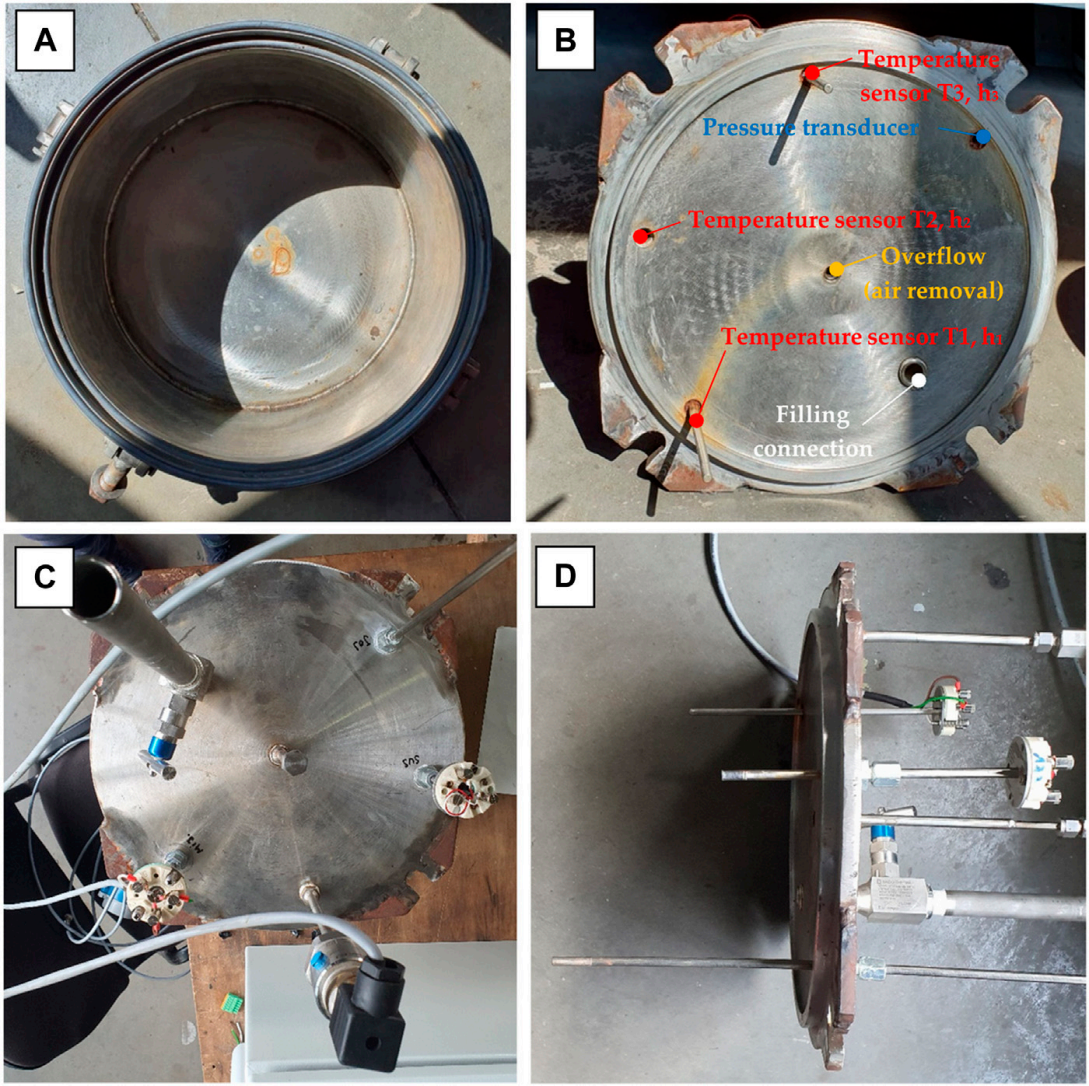
Top view of the isochoric reactor, without the cover plate **(A)**; Bottom view of the cover of the reactor **(B)**; Top view of the isochoric reactor, with the cover plate on **(C)**; Side view of the reactor’s cover **(D)**.

The outer walls of the reactor are welded to four pairs of lugs with straps that make it easier to close the reactor. The reactor cover is made of a 19 mm stainless steel plate (austenitic stainless steel AISI 321 W1.4541 from ITALINOX ROMANIA SRL), having the configuration shown in Figure 2. The reactor cover is attached to the body of the reactor with four M16 × 90 mm screws.

### 2.2 The cooling bath

To reach the desired preservation temperature, a refrigeration device and a cooling bath are needed. The refrigeration system controls the temperature in the cooling bath, and by doing so, the temperature in the isochoric reactor remains constant without major fluctuations. To serve this purpose, we designed a portable 43 L cooling bath with a refrigeration system that can run for days to maintain the temperature constant inside the isochoric reactor.

The cooling bath for the stainless-steel isochoric reactor uses a mechanical vapor compression cooling unit with an air-cooled condenser (Tecumsech AE4440 HR, PS30bar, TS125°C, working with a hermetic piston compressor, Tecumsech AE-8036-BR) and a Ø12 × 1 mm copper coil as the evaporator. The DX cooling machine uses R404A as a refrigerant. The system needs about 40 L of a mixture of 50/50 water and ethylene glycol. To cancel the stratification in the cooling bath, we use a recirculation pump (DAB EVOSTA2, 40-70/130 1”, 3.6 m³/h with a maximum H of 7 mH₂O).

**FIGURE 3:**
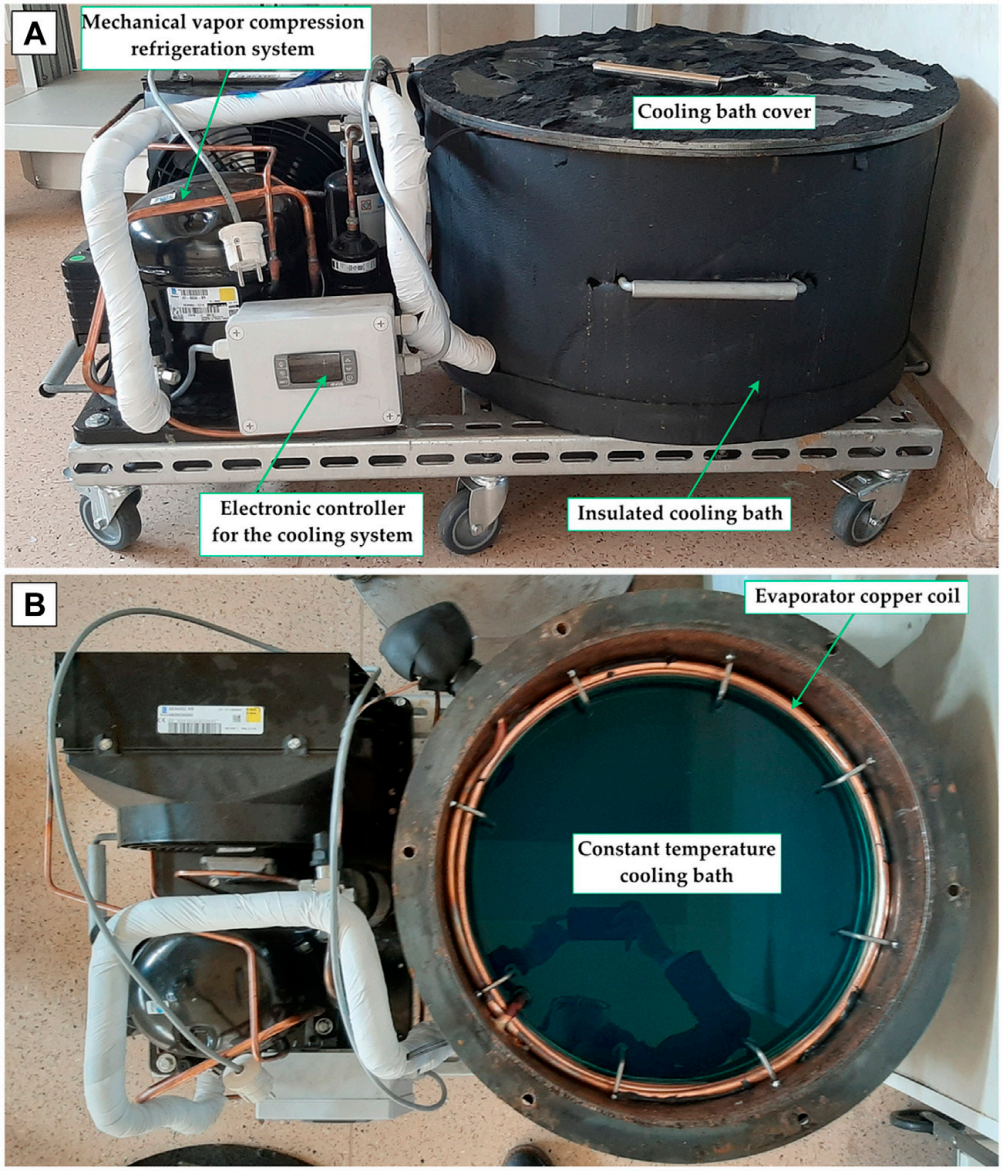
Side view **(A)** and top view **(B)** of the constant temperature cooling bath and the refrigeration system.

The cooling bath and the refrigeration system are placed on a common metal frame equipped with six wheels, to allow the system to be ambulatory. The refrigeration system is controlled by a digital controller with off cycle defrost (Dixell, XR20XC, Emerson Climate Technologies GmbH, Germany), as presented in Figure 3.

When the isochoric vessel is not used for experiments, the cooling bath is protected by a metallic cover provided with a handle.

### 2.3 The pressure transducer

Depending on the desired preservation temperature in the isochoric reactors, the pressure can rapidly increase if isochoric freezing occurs. The reactor described in this paper was designed for isochoric supercooling preservation and can withstand a pressure of 8 bars. In isochoric supercooling preservation pressure is not generated but can accidently occur, in some circumstances. To eliminate the overpressure, a safety head with a rupture disk or a safety valve can be used.

**FIGURE 4:**
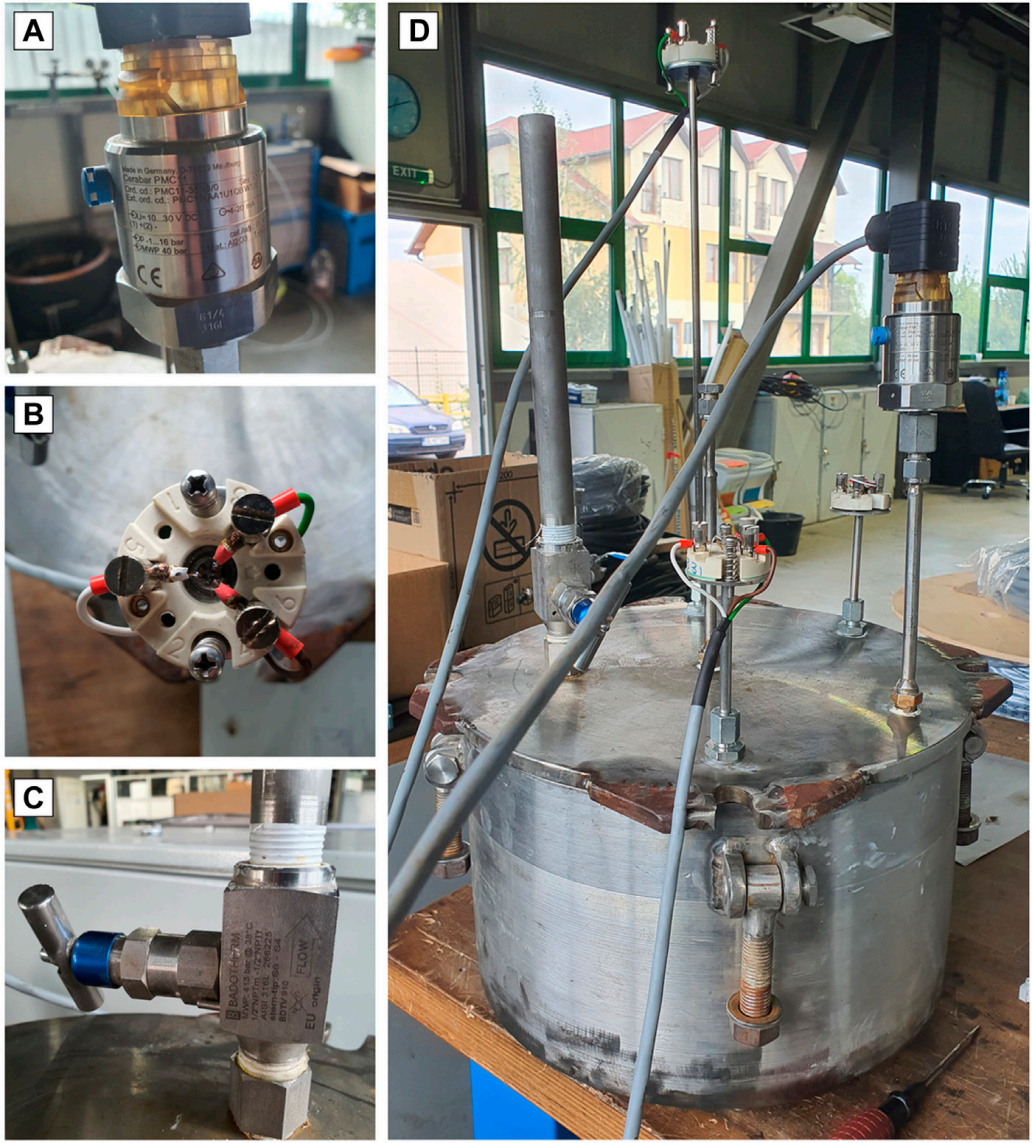
Pressure transducer PMC11 **(A)**; Resistive temperature detectors TR10 **(B)**; Badotherm BDTV910 filling valve **(C)**; The isochoric reactor with all the sensors connected **(D)**.

On this reactor, we installed a Cerabar PMC11 pressure transducer/manometer (Endress + Hauser AG, Switzerland) to measure the internal liquid pressure (Figure 4A). It has a capacitive, oil-free ceramic sensor and is capable of measuring pressure gauge pressure from 400 mbar to 40 bar.

### 2.4 The temperature sensors

We installed three TR10 RTD (Endress + Hauser AG, Switzerland) resistive temperature detectors on this reactor to measure the temperature of the liquid inside the reactor. They can be used in a universal range of applications, can measure between −200 and +600°C (−328°–1,112° F), with pressures up to 75 bar (1,088 psi) (Figure 4B).

### 2.5 The filling valve

The filling connection shown on the cover plate has a BADOTHERM instrument valve (Badotherm BDTV910, EU origin). This valve has a fixed conical tip to ensure perfect alignment, and all parts are made of AISI 316 (L) stainless steel with low carbon content. The process connection is (F) 1/2 ″NPT, the instrument connection is (F1) 1/2” NPT, the maximum pressure it withstands is 413 bar (6,000 psi) at 38°C and the maximum temperature is 240°C (Figure 4C).

The filling line is designed to work together with an overflow connection, so that, when the vessel is filled, the excess preservation liquid will come out. Both, the filling line (380 mm in height) and the overflow connection (180 mm in height) were designed with high heights from the base of the lid, to ensure that the vessel is completely filled, without allowing air to remain in the vessel.

As an additional safety measure, the system can be equipped with a safety valve for overpressure protection. In the present situation, the control of overpressure is done by automatically stopping the refrigeration installation through the automation of the system.

### 2.6 The automation and control

**FIGURE 5:**
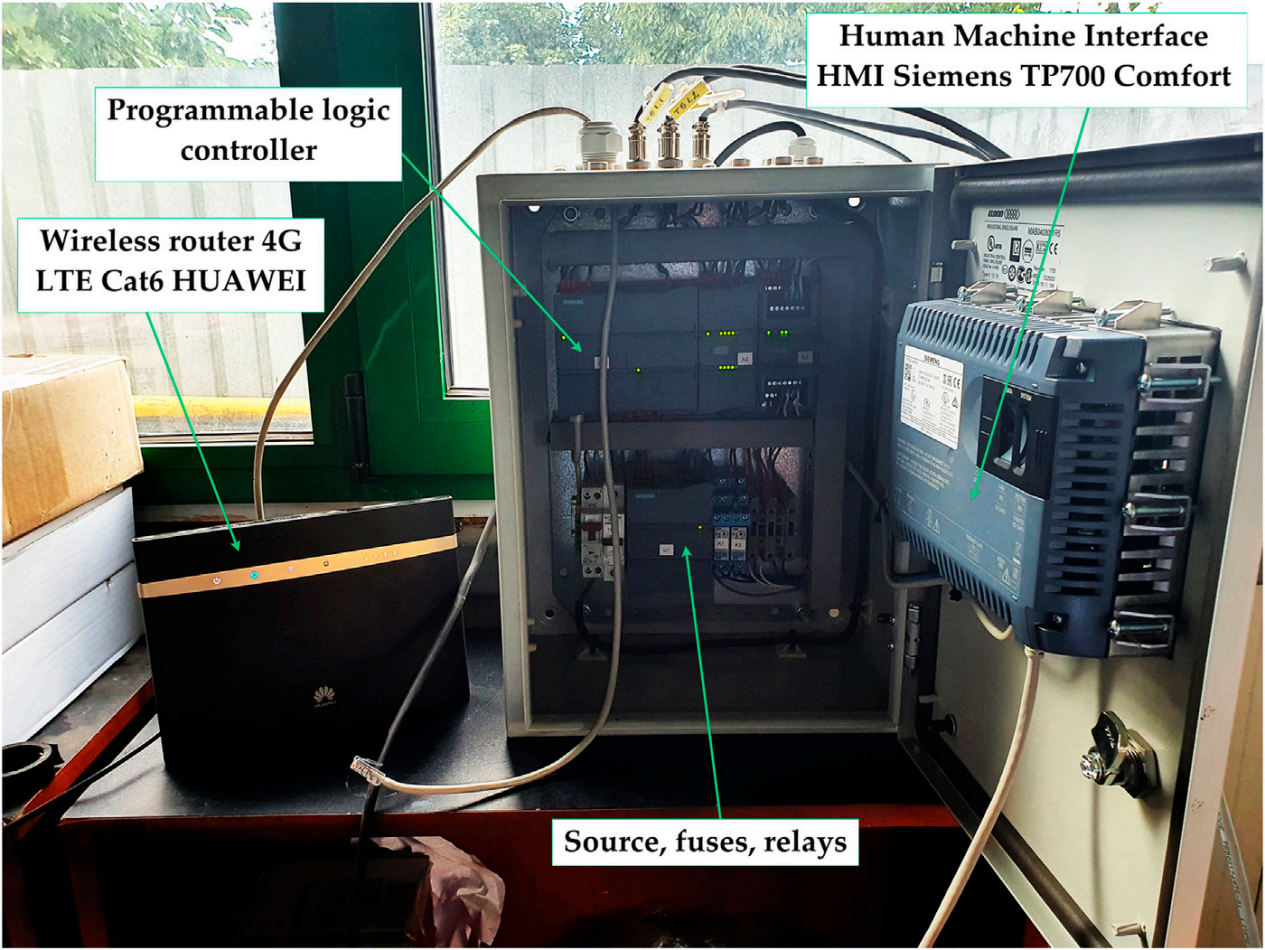
The automation and control panel for the constant temperature cooling bath and the isochoric reactor.

**FIGURE 6:**
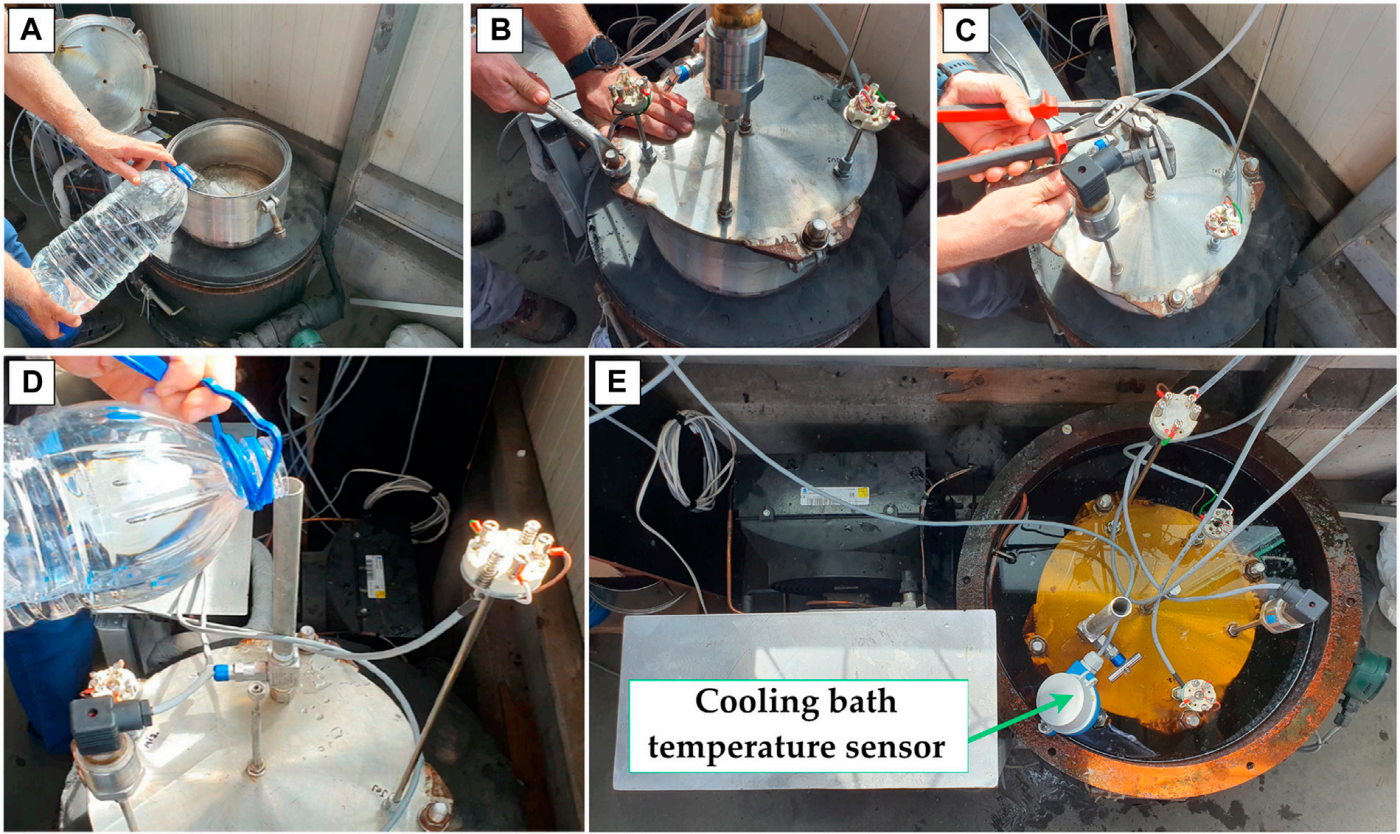
Initial filling with preservation solution **(A)**; Fixing the lid over the isochoric reactor **(B)**; Opening the overflow port **(C)**; Filling with preservation solution and closing the lid **(D)**; Connecting the temperature and pressure sensors to the automation **(E)**.

All devices and transducers are controlled by the control and automation panel shown in Figure 5 and Figure 6, which has three main parts: the programmable logic controller (PLC), sources, fuses, relays, and the human-machine interface (HMI).

**FIGURE 7:**
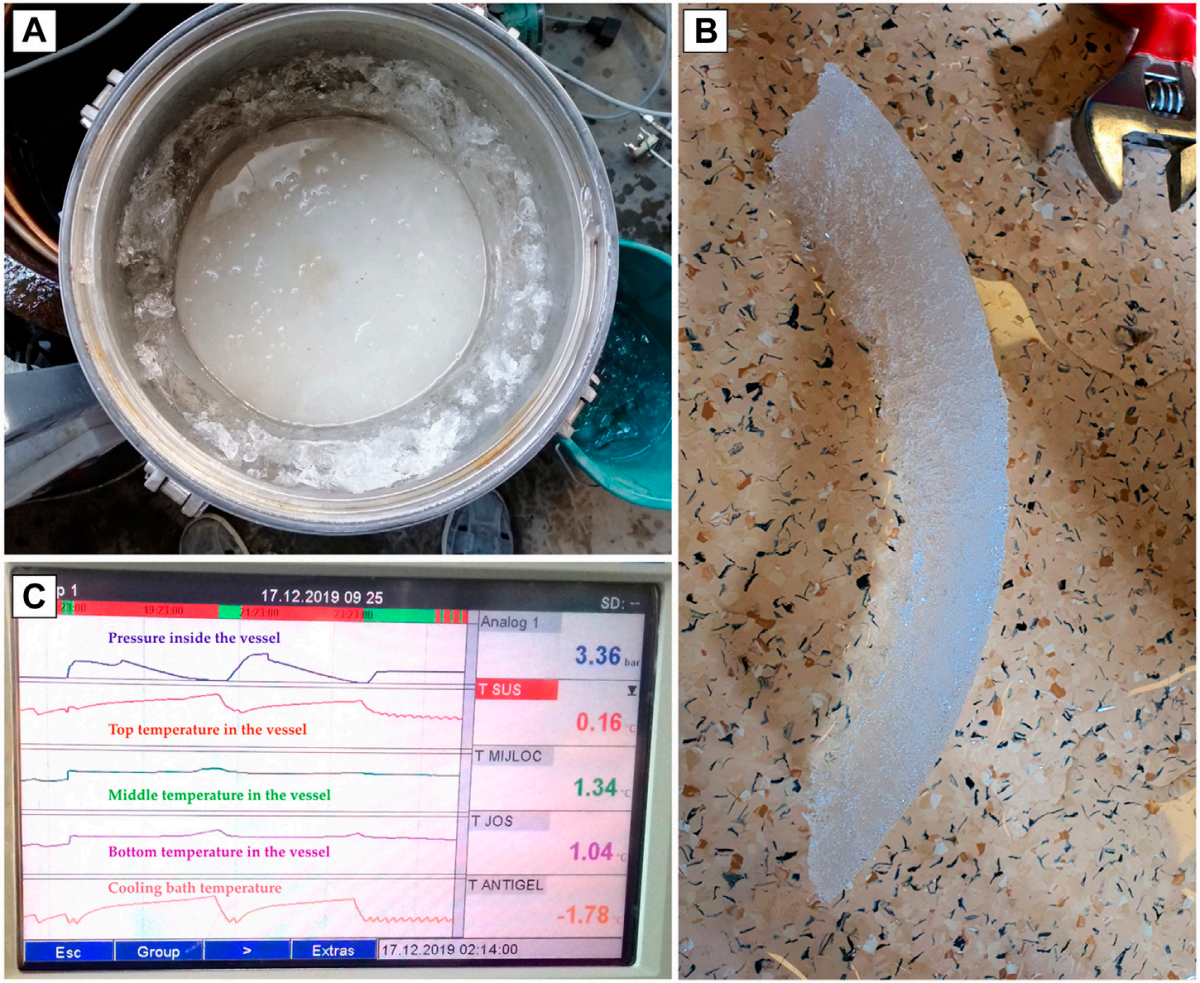
Ice is formed along the walls and in the regions of welding, when isochoric freezing is initiated **(A)**; Ice sheet formed in the isochoric device **(B)**; The touch screen of the human-machine interface **(C)**.

This control panel allows users to remotely control the process over the Internet using VNC and has the ability to store all information on a memory stick or SD card. The human-machine interface has a touch screen (Figure 7C) where users can observe and interact with all the parameters measured in the system.

Because temperature transducers and pressure transducers can act as a “fin” that can lose heat, it is recommended to insulate them with pipe insulation.

To assemble the reactor, we first fill it with the necessary liquid, then the cover plate is placed on top of it and, finally, it is fixed with the help of the four screws and nuts.

Then we open the discharge line and add the filling liquid using the filling line, until it overflows and after we close the instrument valve.

After everything is checked and tightened, the reactor is immersed in the cooling bath and the process is observed on the LCD HMI display, where the data is also stored.

### 2.7 Potential limitations

*Pressure Variability:* the pressure generated inside the isochoric reactor can vary significantly depending on the preservation temperature and the fluid used for preservation, potentially reaching high levels. This variability in pressure could pose challenges in maintaining stable experimental conditions.

*Risk of Ice Formation:* Despite efforts to prevent ice formation through isochoric supercooling, there is a risk that ice may still form under certain conditions, particularly along irregular surfaces within the vessel. This could impact the effectiveness of the preservation process and introduce variability in the outcomes of experiments.

*Experimental Setup:* The design and implementation of the experimental setup, including the isochoric vessel and cooling bath, may appear to be technically complex. As a result, there may be challenges associated with assembly, calibration, and operation, which could affect the reliability and consistency of experimental results.

*Sensitivity to External Factors:* The effectiveness of the preservation method may be sensitive to external factors such as room temperature, radiation, and the insulation of the cooling bath. Fluctuations in these variables could influence the stability of experimental conditions and introduce sources of variability in the data.

*Temperature Control:* Ensuring precise temperature control within the isochoric vessel during preliminary tests may have presented challenges. Variations in temperature distribution or inaccuracies in temperature monitoring could have affected the effectiveness of the supercooling preservation method.

*Equipment Calibration and Setup*: Calibrating and setting up the experimental equipment, including temperature sensors, pressure transducers, and cooling systems, may pose challenges. Ensuring accurate instrument readings and proper functionality of the experimental setup is critical for obtaining reliable results.

*Optimization of Experimental Parameters*: Determining the optimal experimental parameters, such as cooling rates, preservation temperatures, and pressure thresholds, may have required iterative testing and optimization. Identifying the most effective conditions for supercooling preservation is essential for the success of such experiments.

*Safety Considerations*: Addressing safety considerations, such as the handling of high-pressure systems and potential risks associated with supercooling processes, may have been a challenge during preliminary tests. Ensuring the safety of researchers and the proper handling of experimental equipment is paramount in laboratory settings.

This was also the purpose of this prototype, namely, to see what are potential limitations of such a technology, by trying the system in different conditions to cancel or reduce these limitations.

## 3 Experimental protocol

Before assembling the components, they must be perfectly clean. They are cleaned with water and wiped with dry paper towels, both inside and out.

When assembling, two rubber O-rings Ø4mm are used for sealing, and the components are tightened with a turnkey, always keeping in mind that the pressure that the system can reach, at equilibrium varies, depending on the temperature, but this can also be 2000 bar or over under certain conditions.

Because the temperature transducers and pressure transducers can act as a “fin” that can lose heat, it is recommended to insulate them with pipe insulation.

To assemble the reactor, we fill it first with the necessary liquid, then the cover plate is placed on top of it, and in the end is secured using the four screws and nuts.

Then, open the overflow line and add top up liquid using the filling line, until it overflows and after we close the instrument valve.

After everything is verified and tightened, the reactor is immersed in the cooling bath, and the process is observed on the HMI LCD display, where data is also stored.

1. Switch on the cooling bath and set the desired temperature for the experiment. The duration of the target temperature depends on the room temperature, the presence or absence of radiation from various sources (for example, solar radiation during the summer) and how well it is insulated on the outer wall of the cooling bath. There must be a mixture of water and 50/50 ethylene glycol in the cooling bath to be able to reach negative temperatures of down to −30 °C.
2. As long as the cooling bath cools to the desired temperature, the experimental solution may be prepared, if necessary, or precooled in the refrigerator, if necessary. When using water as a working fluid, it is recommended to use distilled water with a neutral pH.
3. Fill the isochoric vessel with the solution from the experiment. Because air bubbles can appear in the solution and the air is compressible, unlike liquids, it can cause many measurement errors, so after filling the reactor, it is recommended to pour the liquid out of the container to remove any air bubbles.
4. Tightening the container in the pressure transducer is sensitive, as it is desired that the initial pressure of the experiment be about 1 bar or less. Please note that forcibly tightening the two elements can generate static pressures of up to 50 bar inside, leading to erroneous data from the beginning.
5. Place the isochoric system in the cooling bath, with the transducers facing up, but not completely submerged, but only in the connection area, to avoid contact of the electrical parts with the liquid.
6. It should be noted that three experiments must be performed, and all three experiments must obtain the same values, otherwise the experiment is not valid. This is done either by repeating the experiment three times, or using three isochoric systems simultaneously.
7. At the end of the experiment, remove the isochoric system from the cooling bath and leave it at room temperature or heat it to room temperature until the pressure indicated by the software is 0 bar.
8. If only one isochoric system is used in the experiment, repeat all the steps presented three times, until three similar graphs are obtained for each experiment studied.
9. After completing all experiments, the data acquisition is stopped and the data is exported to EXCEL for post-processing. At the same time, the cooling machine connected to the cooling bath also shuts down.
10. All components are disassembled, thoroughly cleaned with water, wiped with paper towels, and stored in their original place. The solutions used in the experiment are thrown into the sewer, and their containers are closed and placed in their original place and position.

Because we deal with an isochoric device, if the preservation liquid nucleates and ice formation is initiated, then the supercooling state changes into isochoric freezing. Our research team has much experience with isochoric freezing, but generally with small-volume vessels. In an isochoric state, the vessel needs to withstand the pressures generated by the ice formed inside, but this way, it will end up with a very large and heavy vessel. So, it is important to have a method of control that can stabilize the supercooling state.

There are two essential elements that need to be carefully controlled in isochoric studies: the elimination of free air from the system and the initial pressure within the system.

In our preliminary tests with distilled water, the ice formed along the walls and in the regions of welding, when isochoric freezing was initiated. This suggests that ice formation was induced by the inner irregular surfaces of the vessel.

## 4 Conclusion

The technology developed in this study is new. This prototype is the first to pursue an in-depth and systematic study of the concept of organ preservation using isochoric supercooling. Therefore, we are advancing carefully, and we first optimized the design of the devices from this study (Năstase et al., 2023). We checked every aspect of the devices–prior to animal experiments. After the devices are found to work satisfactorily in experiments without biological matter, we will test their performance on pig livers. These tests will be for the performance of the devices. After we confirm that the devices work as designed, we will begin *in vivo* experiments. The experiments will be supported by mathematical studies and designed with parameters that we anticipate will yield optimal preservation. After each experiment, our team will gather and analyze the results, designing the new experiments on the basis of the findings of the previous ones.

The future directions of our research aim at creating a more compact, cheaper, lighter, and portable version of this technology, while contributing to advancements in the field of organ preservation and addressing real-world challenges and needs.

In this study, we present the design and concept of a large isochoric container and the refrigeration machine that can be used to supercool a fluid. The ambulatory system can be used for cryopreservation and transportation of whole organs, in a supercooled state, in isochoric conditions. The preservation of organs poses many challenges, because an organ is a complex biological system that consists of a collection of tissues that perform a specific function. A successful preservation of an organ by supercooling might be the answer for all the patients in need.

## Data Availability

The datasets presented in this article are not readily available because of restrictions. Requests to access the datasets should be directed to gabrielnastase@unitbv.ro.
